# The Privileges and Duties of the Members of the American Society of Dental Surgeons

**Published:** 1845-06

**Authors:** 


THE AMERICAN

### JOURNAL OF DENTAL SCIENCE.

Vol. V.] JUNE, 1845. [No. 4.

### ARTICLE I.

***The Privileges and Duties of the Members of the American Society of Dental Surgeons.*** **By the SYRACUSE EDITOR.**

HAD caries in teeth never been successfully arrested, or a diseased mouth restored to soundness, or, in short, any of the thousand evils incident to a diseased denture, either mitigated, cured or averted by dental practice, the idea of *quackery* in dentistry would be paradoxical. We may, therefore, to some extent, estimate the value and claims of skilful dental operations, by the swarms of empirics which infest our land, and who affect to dispense the benefits resulting from scientific practice. We may also infer, from the same observation, the extent and magnitude of the evils calling for such aid. Indeed, were we to determine the true standard of either, by adopting *this* rule, we should give to the profession a greater elevation, and to these ills a more aggravated character, than is claimed by the greatest stickler for the honor, the dignity, and the claims of this most powerful auxiliary to the general art of healing.

And it is not a little surprising, that any profession, whose resources are so abundant, and whose benefits so great and palpable, could, in the present enlightened age, be so distorted and defaced by imposture and empiricism. For, although the profession of dental surgery can boast of its thousands of living witnesses, who would cheerfully testify to the great and inestimable value of her gifts, it is, nevertheless, true, that at this very moment, a large majority of all the inhabitants of the United States, either discard wholly her claims, and set her professors among the many which expect to succeed only by deception, or consider such claims so doubtful, as to regard an experimental test, as involving more hazard than would be compensated by any possible benefit which they expect to realize.

This aspect must be exceedingly painful, not only to the worthy members of the dental profession, but to the philanthropist who has a just appreciation of the great benefits which dental science is capable of bestowing upon the human race, it not only involving the character of the profession itself, but also the honor of its members, and depriving thousands of those blessings which should be common to all.

We need not add, that this low standard of merit attached to dentistry by so great a share of the public, has been fixed by the malpractices of ignorant pretenders.

The community have judged of the tree, by the only fruits which they have seen, and have passed sentence accordingly. Now, what high-minded, honorable dental surgeon, on viewing this state of things can but feel a willingness, nay, a strong desire to put forth his utmost efforts to strip his profession of these clogs, and to assist in placing it on that eminence, which, by its intrinsic merits, it may justly claim? Indifference in this matter is, to us, an incomprehensible feeling, and can be explained only on the ground of a total destitution of all those higher endowments which make the distinction between man and the lower orders of creation, or that every ray, from every great and noble object, is intercepted and absorbed by the sixpence which may intervene.

Now, are the evils here spoken of, susceptible of remedy? and if so, who are to be the agents of the required reform, and what mighty engine shall be employed to effect this great work?

In presenting some considerations bearing upon these consecutive questions, it may be remarked that the hope of elevating any profession, and of freeing it from abuses, must bear a direct ratio to the practicability of reducing it to definite limits, and clearly defining the principles upon which correct practice is to be based. For example, in the general practice of medicine, the field must ever be subject to the inroads of quacks, without the possibility, on the part of its worthy practitioners, of demonstrating to the world the falsity of the doctrines or practice of the merest charlatan. This arises, partially, from the fact that such practice is frequently, with the most learned of the profession, necessarily based upon theories, vague and unsatisfactory even to themselves, and which, of course, cannot be rendered otherwise to the public. Nor is it less difficult to disprove a theory of a rival competitor, which, however false it may be, is sufficiently ingenious to deceive those unacquainted with the labyrinth of sophistry. Hence appears one sect, declaring that all disease is occasioned by a deviation from a certain standard of heat or cold, and that every remedy, to be available, must be prescribed and administered in accordance with this principle. Another, claiming that every deviation from health arises from local irritation; and another, that the whole should be ascribed to general irritation: and so on, till a long list of these erratic planets have, at different times, arisen and set, each eclipsing and in turn being eclipsed. Again, one class have deemed it expedient to use remedies from each of the three kingdoms of nature, the mineral, vegetable and animal; another have contended that all mineral medicines were necessarily poisonous, and hence should be stricken from the list of remedies. But although the views of these two have been antipodal in respect to *kind* of remedies, yet both agree that an appreciable quantity is necessary, while a third, claiming equal skill, advocating the doctrine of infinite divisibility, contend that the effect of any remedy in overcoming disease, is directly in proportion to the minuteness of the dose.

Another consideration worthy of notice, and which tends to perplex the public mind, and render it difficult for them to discriminate between true and false theory, is the fact that the physician deals exclusively with organs capable of *self-restoration;* the patients frequently not being able to determine whether they recover, if at all, in consequence, or in spite of the remedy. It is by virtue of this principle, we have no doubt, that some of the theories and practices which have been, from time to time, more or less popular, have been tolerated. But it is foreign to our object to meddle with medical theories, farther than to illustrate the difficulty, or indeed the utter impossibility of having a uniform standard, or of creating universal confidence in any profession, whose theories and practice admit of such great diversity. Fortunately, neither of these obstacles, so formidable in medicine, pertain in any considerable degree to the profession of dental surgery.

There is, perhaps, no profession, certainly no branch of the healing art, in which the fundamental principles on which its utility is based are more susceptible of demonstration, than in dentistry. Upon nearly every doctrine, serving as the foundation of dental practice, are the intelligent members of the profession agreed; and it may be remarked, moreover, that there is very little room for doubt whether any given result of an operation is attributable to skill or the want of it, or whether the same result would have obtained, had no treatment been adopted. Hence, it only remains to disseminate correct notions and correct practice, and we may have the cheering prospect of a certain victory.

Assuming, then, that there is certainly no insurmountable obstacle to the freeing the dental profession of its abuses, we proceed to inquire, who shall be the *agents* of this reform? Upon this point little need be said, as it must be plain to all that this task devolves alone upon the members of the dental profession. They only can understand the extent and nature of the evils to be remedied, and unless they succeed in purifying their own ranks, the task will remain unaccomplished. Some assistance might be expected from physicians, but we are fully satisfied if any aid be derived from this source, it must be regarded as simply and strictly auxiliary. They cannot be expected to lead in the enterprise, as this would involve a knowledge of the dental profession which they by no means possess.

If, then, this reforming task must devolve upon the members of the dental profession, let us again inquire, with what vast engine are they to accomplish this great work? Upon this point, let us consult the spirit and the successful enterprises of the age, and see whether we may not from these sources derive some important instruction. Than the age in which we live, none has ever been more signalized for great and important discoveries and achievements in every department of art, science and literature—none more, for great and successful moral enterprises; and none, in short, has ever been so characterised for those rapid and powerful strides of mind, in the accomplishment of the most magnificent undertakings, either in themselves considered, or in reference to their results. And what age, we inquire, has been so remarkable for *voluntary associated effort*?

Nor do we regard these results as merely incidental, but as the legitimate operation of cause and effect. While on the one hand *such effort* has shown itself far more efficient than the same amount of individual effort, it may also be truly remarked, that as intelligence progresses and becomes general, assimilation of thought, feeling and desire necessarily advances, till almost the inevitable result is, a uniting of forces, with a view to overcome both physical and moral obstacles. And the resistless momentum of such a combination of living forces is beginning to be felt and acknowledged, wherever their agency has been applied. The results of the efforts of many of these voluntary associations will ever be looked upon as the most splendid achievements of modern times. Their influence will extend through all time, nay, to the boundless ages of eternity. And it should be borne in mind, that this tendency to association is the necessary result of intelligence.

That ignorance is decidedly anti-social, the history of the past most clearly proves. But let us mark this effect in a few individual instances. The time is not far in the past, when first in our country the intelligence of the people decided the necessity and practicability of the great internal improvements, which have so many fold increased its resources. Where but yesterday were only seen the unbroken forest, the rugged mountain resting firm on its rocky foundation, the impassable swamp, the deep ravine, the unbridged river, is now the firm and even rail-road track, over which passes, hourly, the beautiful car, with its nimble wheels, filled with passengers of pleasure or business, or laden with the rich products of a prolific soil. These great thoroughfares have, in effect, annihilated time and distance, made contiguous distant cities, and brought the most available marts within the immediate vicinity of every portion of the entire country. Nor have these facilities for rapid communication been less efficient and beneficial in disseminating intelligence, useful knowledge, refinement and the thousand blessings which had before been comparatively confined to particular portions. In short, they have converted the vast multitudes, occupying a still more vast extent of territory, into one great community, where each may enjoy all the privileges, blessings and resources possessed by the whole. But suppose that these works, so magnificent in themselves, and so vast in their consequences, had been undertaken on the plan of individual enterprize, and that each individual had attempted to construct such a portion of our rail-roads and canals as was compatible with his ability or inclination, what must have been the result? Instead of having one unbroken chain of communication, connecting the most remote parts of our vast country, resembling the current of circulation, by enabling each portion to impart a salutary influence to all the rest, and, in turn, to be strengthened and invigorated by the whole, we should have only isolated portions, serving no good purpose—the whole scheme ending in total defeat, loss and disappointment. But mark the result when the engine of associated effort heads the train. The whole work springs into existence as by magic, and every natural resource is at once brought under the control and into the service of man. Do we need proof of the efficiency of associated effort in carrying on great moral and religious enterprises, in disseminating knowledge and refinement, and in eradicating, not only those evils which are the legitimate offsprings of ignorance and superstition, but those, also, which have ever been the concomitants of wealth and luxury? If so, we have only to point to the different associations formed to accomplish these several objects, and the results. Where but a few years ago, millions of rational, accountable beings were in the lowest state of degradation which ignorance, paganism, and an unlimited sway of depraved propensities could place them, are now, as many, enjoying the innumerable and inestimable blessings of christianity, and a comparative degree of civilization. Who can but admire the silent potency of means accomplishing thus rapidly such great ends? and our wonder and admiration will be much enhanced by reflecting that the field of operation has generally been thousands of miles from the vast majority of the laborers.

The youngest of us may remember, when, in our own happy land, the tender of *cordiality*, or even of *civility*, was by no means complete, without the intoxicating bowl; nor was this custom confined either to age or sex.

He must remember, too, another feature inseparably connected with this universal fashion, which, we doubt not, he would fain forget; the degradation and vice, and misery, which had exhaled from this fashion, which, like an atmosphere surcharged with infection, was destroying, in its course, its thousands of victims by a death more terrible than the plague itself. Although, wholly to eradicate the overwhelming evils of intemperance, much remains to be done, yet the great change which has been already wrought is truly surprising to all who have duly considered the strength of the fetters which this habit rivets upon its victims, especially when their deformity was hid, and their clanking hushed by the silken robe of fashion, till this fairy creature refused to follow farther, on their road to ruin, those whom she had crushed, but whose loathsomeness her gaudy vestments could no longer hide. Nor can the "causality" of any be so dormant as not to inquire the means used to effect so difficult a reform, nor his "veneration" so feeble, as not to be forcibly struck with the efficiency, and yet simplicity of the agency employed. *Associated effort*, in giving the right direction to public sentiment, has been the mainspring and prime mover in accomplishing the whole work.

But we need not particularise farther to establish our position: suffice it to say, that whenever any great or extensive work was to be accomplished, good effected, or evil eradicated, the task has been, by common consent, assigned to associations.

It was this confidence in the powers of association, which inspired the "first few," whose names, for generations to come, will be "of happy memory," to undertake, by formation of the American Society of Dental Surgeons, the great work of reforming the dental profession; of elevating its standard, and of suppressing its quackery. Nor do they now need the inspiration of analogy to give them the fullest and most sanguine hope of ultimately meeting with entire success. As in most untried experiments, whose success would involve, to some extent, the foresight and reputation of the projectors, few, comparatively, were ready at the outset to raise the standard, and declare their intentions to stand by it.

But since its first meeting, which was August, 1840, this society has increased at least tenfold, to say nothing of its honorary members. Although the increase has been rapid, including many of the first men in the profession, yet we have good reason to believe that few, comparatively, of all who *should* lend their aid in forwarding this noble enterprise have yet come to the rescue. Of the zeal of the members now composing the society, in the main, we have no reason to complain, although we feel that there are some exceptions, and to these, before we close, we shall have a word to say. "The object of this society" being "to promote union and harmony, among all respectable and well informed dental surgeons, to advance the science by a free communication and interchange of sentiments, either written or verbal, between members of the society, both in this and in other countries, in fine, to give character and respectability to the profession, by establishing a line of distinction between the truly meritorious, and skilful, and such as riot in the ill-gotten fruits of unblushing impudence and empiricism;" and it being pretty clearly demonstrated, both, by reference to similar associations, to effect similar reformations, and to the present aspect and influence of *this* society, that this association may be fully adequate to accomplish the desired end, we shall offer some reasons why every professed dentist, who is not a quack, should become a member, and why every member should attend its conventions as often as practicable, and in all proper ways seek to promote its success in the accomplishment of these desirable objects.

In addressing those who are not members of this society, and whom we would persuade to become such, we shall suppose them, for convenience sake, divided into two classes; the former, including those who have an established reputation and practice, and whose *interest* hence, we will suppose, is not to be promoted by any reflux influence of this association; the latter, younger practitioners, who, though intelligent, have yet their reputation, their practice, and their fortunes to acquire.

What motive, then, we inquire, have those embraced in the former class, for putting themselves to the trouble of forming and supporting societies which will not augment, in turn, their own prosperity? Point me to the man who claims that with *him* no such motive exists, and we will show you one, who might, with the greatest propriety, *also* claim, that if he could but reap a golden harvest from his vocation, it mattered not whether it was honorable or dishonorable, whether it was productive of good or evil, or whether his calling sustained an elevated rank among honorable professions, or sunk into utter disgrace in the hands of the detestable empiric or the ignorant and dishonest quack. Such an one could, doubtless, listen with *indifference*, if not with a relish, to the taunts and scandal so frequently lavished upon the whole fraternity, in consequence of the malpractices of ignorant pretenders. But we need not enlarge upon the infinite contractedness of such sentiments, or the unenviable disposition of those who entertain them, as we neither expect nor wish such to join our ranks.

But we have good reason to believe that a great number of highly intelligent and honorable members of the profession are still without its ranks, and for some reason choose to stand aloof from it. Now, although we may assume that by their co-operation with this society, neither their reputation as honorable men would be augmented, nor their pecuniary interest enhanced, yet it seems to us that there are other and higher claims which each ought to regard as binding upon himself. Simply because one is independent of any direct gain which he may derive to himself, is he the less interested in promoting the general good and welfare and elevation of the profession? Is he less interested in freeing it from the stains which empirics have sought to make indelible? and if not, what excuse has he for either refusing or neglecting to employ those means which alone promise to bring about so desirable an end? If the profession at large is to be benefitted by the operation of this society, surely each member must be a partaker, and shall we suppose any one willing to enjoy these benefits, while he stands an idle spectator—willing to be floated along to prosperity and eminence, while his brethren are making their utmost exertions to propel the bark against the strong and impetuous current of empiricism? Surely, no honorable, high-minded dental surgeon could remain in indifference; and if not, what time can he embrace more favorable than the present, to set his profession upon an elevated stand? The present crisis is, moreover, one in which his aid is strongly demanded. The standard has been raised by the American Society, to a height which must meet the views of the most scrupulous; and many of the first men of the profession have gathered around it and resolved to stand by it.

Empirics have already taken the alarm, feeling that "their craft is in danger." Hence, all who are desirous of breaking their ranks, are now called upon, not only to come out from them, but publicly to take up arms against them. Were every honest, intelligent dentist to draw this line rigidly, the victory would be both certain and speedy. Although there might be a great disparity in numbers, yet "one man," thoroughly imbued with the love of science, "would chase a thousand" of these charlatans, "and two put ten thousand to flight." If then, there is every reason to place great confidence in the associated efforts of the worthy members of the profession, to place dentistry upon its true basis, and free it from the odium which impostors have attached to it, we again inquire what excuse can any one, claiming respectability, offer for withholding his influence, though it require some personal sacrifice. Do any object that the organization of the American Society of Dental Surgeons is imperfect, and that it is, hence, unworthy of confidence? If so, we would remind them, that both the constitution and by-laws are subject to any alteration which experience may dictate, and that these alterations can be made only by members of the society. Several have already been made, since its adoption, chiefly relating to the admission of members. It has ever been true that those societies, which justly gave distinction to their members, have found it necessary to guard against the admission of those who seek membership for the sake of that distinction which their qualifications and intrinsic worth had failed to secure. The just apprehension of danger from such a cause, was fully felt by the original founders of the society; nor has the vigilance, in respect to this point, which, at the outset, was regarded the only guarantee of success, been at all abated. Nay, this conviction has waxed stronger and stronger by the experience which each succeeding year has brought to the aid of its members, in fixing the proper restriction for membership. Hence, in addition to the original guards, new and more efficient measures have, from time to time, been adopted, not only to protect its future growth, but to prune from the trunk every noxious or unfruitful shoot. With the constant exercise of this judicious care and vigilance in admitting only worthy members, not a doubt need be entertained as to its final success and triumph. Let but the American Society of Dental Surgeons see to it, that she has no enemies within the camp, and she need fear no foes without. If any then object to lending their aid in carrying out the objects of this society, because they, "with purer eyes see imperfections," we ask them to come into its ranks, and we will assure them the most ample scope for the full exercise of *all* their powers in the work of purification.

These men so ardent to raise their professional standard to a spotless perfection, and so eagle-eyed, in discovering the faults and frailties of others, are the very men (doubtless, in their own judgments) needed at the helm.

And they may receive the utmost assurance, moreover, that they will be promoted to that responsible station, on giving satisfactory evidence of their ability to steer the ship to the desired haven. But if, on the other hand, those who urge this kind of objection still choose to act the *Pharisee at a distance*, we cannot refrain from suspecting their sincerity, and shall be strongly inclined to look farther for the real cause of repulsion.

If this ground does not warrant the imputation of quackery, it certainly lays upon them the charge of exerting a most unwarrantable and pernicious influence; inasmuch, as it makes a broad and impenetrable shield for every empiric, and speaks a language which is echoed by the basest imposter with the most enthusiastic delight. If there is any one object which, above all others, the dental quack would delight to see perfected, it is the destruction of the American Society of Dental Surgeons. He looks upon it as a monster, created to usurp his right; and its overthrow, regardless of the means, is with him a darling theme. To effect this, he chiefly relies either upon the imaginary, or perhaps, in some instances, the real faults of its members, and upon the taunt which he too frequently hears from those whose example should be to him like the touch of the torpedo, instead of encouraging him in his work of destruction.

Thus far we have embraced, in our remarks, that class of dental practitioners, who have so far ascended the hill of dental science, and were so advanced on the road to wealth and eminence, as to derive no direct benefit from any aid which the society could, in turn, bring them for their efforts to promote its interests and objects.

It may be objected, however, that individuals who would strictly come under this description are far less numerous than might be at first supposed. Nothing is more certain than that the American Society contains men, not only of the first order of talents, and of acquirements, but also men of wealth, yet we feel fully warranted in asserting, that the most elevated among us have been many fold repaid, by the direct reflex influence from this source. It seems to us remarkably paradoxical, to suppose that *any* man is so very learned, or skilled in the art and science of dental surgery, as to meet, annually, a hundred of the first men of the profession, all intent on gaining and imparting information, respecting their favorite profession, and the best means of elevating its standard, and promoting its interests, and still go away unbenefitted, and feeling that his time has been misspent. It is equally paradoxical to believe that any *can* claim so much excellence and eminence, and can have thrown such a halo about *his* practice as that all successful means to elevate the profession at large, and suppress empiricism, could in no wise benefit *him.* This aspect of the case leaves entirely out of view that pleasure, and genuine satisfaction, which men of generous mould ever feel from the consciousness of having contributed by their efforts to the advancement of a good and noble object. We come now to address some remarks to the second class or division which we alluded to in the commencement, viz. those whose eminence was not such as to render them independent of the benefits to be derived to themselves, by a union with the American Society of Dental Surgeons.

This class embraces all those whose aims are high, but who are still desirous of augmenting their fund of knowledge, and of rearing for themselves an honest reputation. While, by securing membership, the junior members may enjoy, in common with the seniors, all the general benefits to be derived from the elevating of the profession, and the suppression of quackery, and from the consciousness of being the instruments of reform, they have additional inducements, which are less, and in some cases, possibly, not at all applicable to the senior members. Among these may be enumeratd the enhanced facility of acquiring correct notions of correct practice, which alone will serve as the basis of a valuable and lasting reputation. Hitherto there has existed in the dental profession, a jealousy which has proved an impassable barrier to that free communication and interchange of views so necessary to the advance of its true interests, and the dissemination of useful practical information. As a necessary result, the younger members who were *anxious* to improve, come up to the proper standard of qualification, were, in many and in most instances, for want of a proper passport to confidence to those competent to instruct them, obliged to content themselves either with the result of their own experiments, or that imperfect description which books alone can give in mechanical and operative dentistry. Many have thus become discouraged and settled down in quackery, whereas, they might, from their talents and from their original desire for knowledge, have become ornaments to the profession, had this love not been blighted by the cold, not to say unprofessional, or even ungentlemanly treatment of those they looked up to as their superiors. The mere fact that one introduces himself as one of the fraternity, has often been an excuse for that treatment which would have been justly regarded as incivility, extended to a beggar asking alms! This is radically wrong, and is calculated to make a most unhappy impression upon the public mind, and results mainly from the want of a standard by which to graduate the merits of every practitioner. Dentists have been fully aware, that thousands having no qualification but impudence were constantly

"Prowling each place still in new colors decked,"

seeking just that amount of information which would enable them to impose most successfully upon the public and the profession. Every stranger was hence suspected and treated accordingly. This course has found its excuse in the belief, that it was the only available safeguard against imposition. While this, in many instances, has been too true, yet it certainly exhibits a state of the profession calling most loudly for a remedy. What shall be that remedy? Before endeavoring to settle this question, let us, for a moment, inquire more particularly into the cause of the evil. Why then has a dentist denied to a brother that hospitality which he is wont to bestow upon others? Can it be simply because he is engaged in the same profession? This certainly cannot be the explanation. No, the secret lies in the fact, that he fears he may be an impostor, and dares not be frank with him, lest his frankness be turned to his own disadvantage. If this view of the subject be correct, it must follow, that any measure that would serve to create a proper confidence between the different members of the profession, would take away this barrier to free communication, and remedy the evil. All that is needed then is, a test of qualifications that is reliable. This every member of the American Society of Dental Surgeons has, and may, without fear, exhibit, as incontestible proof, that he is regarded, by good authority, as a worthy practitioner of dental surgery.

The following case, for the correct statement of which we can fully vouch, may serve to illustrate not only the feeling which too commonly existed between members of the dental profession, and which, we are sorry to add, is far from being wholly extinct, but also the effect which the American Society is capable of producing; substituting for that miserable spirit of distrust and jealousy, a brotherly regard, so desirable among all worthy members of the same fraternity.

In the winter of 1839, Mr. A. a young gentleman, a graduate of one of our most respectable medical colleges, who had, for several years devoted his attention to dental practice, called upon Mr. B., a dentist of reputation, in one of our cities, to whom he had taken pains to obtain letters of commendation, with the view of gaining information in the profession of his choice, and which he strongly desired to cultivate. On presenting his letters, and making known his business, he received the following laconic reply, in a tone which could not be mistaken: "If you will pay me $250, cash in hand, and give me an endorsed note of $250 more, you may come into my office, and stay as long, or as *short a term*, as you please."

The spirit here shown needs no comment. This requisition being entirely beyond the limit of the young man's means, he returned home disheartened, if not disgusted.

But he was not to be baffled in his determination to excel, but with a zeal which we are happy to say has not proved wholly barren, set resolutely about making the most of the means which were at his command. These consisted in the dim light of books, and the experience gained by his own experiments, which were but too often failures. Thus, for two years he went on as fully intent on his object, as was ever an alchemist on finding the philosopher's stone.

At length the *American Society of Dental Surgeons* struck his vision, as a new and attractive luminary. He lost no time in availing himself of the benefits which it proffered to all who were qualified to receive them, and who were willing to make the necessary sacrifice, to gain the true path to professional eminence. Nor were his most ardent hopes disappointed. Here, instead of meeting that cold reserve, so calculated to turn day into night, and extinguish every better feeling of the heart, he met frankness and kindness, and that full flow of fraternal cordiality for which his soul had so long panted. So sudden, and great was the change, that he could hardly believe himself an inhabitant of the same earth, and, at intervals, almost feared the transport an illusion too joyous to be real. Well may we believe these his emotions, when we consider, that for him to be thus surrounded, by a hundred high-minded, honorable men, whose faces glowed with intelligence and kindness—each anxious to receive, and willing to impart, that knowledge which it had been the work of years to collect—drawn together from the most distant parts of the country by a common object, whereas, but yesterday, he met only the frowns of bigotry, and the scorn of suspicion. But thrice happy was he when he found this was no dream, no illusion.

He returned from this annual convention, filled with new hopes, enlarged views, and strengthened resolution, to do all in his power, not only to add to his thus greatly increased fund of knowledge, but to make any sacrifice, consistent with his ability, to elevate the profession, and free it from those clogs which had so greatly impeded its advance. This to him was, emphatically, a new era, a day of jubilee. He now not only constantly rejoiced in his multiplied sources of improvement, but still more, in the hope of one day seeing his favorite science elevated to its just rank among the other learned professions.

Thus, through the succeeding year, his labors were refreshed, and his toils sweetened; looking forward to the happy time, when he should again meet, and greet, those who had done so much to enhance his pleasures, and to brighten his prospects. The year rolled round, and though a journey of nearly a thousand miles intervened, he found himself, at the roll-call, again in the midst of those to whom he so reluctantly bid a temporary adieu, a year before, and from whose counsels he had derived such great benefit.

At this session he met many who were not in attendance the previous year, and among these he recognized Mr. B., the dentist to whom he had before applied for instruction. But he soon discovered that he was not recognized in turn. He, therefore, sought and obtained a formal introduction, with the determination to renew his request for instruction, with the view to learn whether his *relative position*, would or would not secure better success.

With this view, therefore, as soon as a convenient opportunity offered, he addressed him, in substance, as follows: "Sir, from the opinion which I have long entertained of your *operations*, I have been desirous of learning your method of overcoming many difficulties which have been to me sources of great perplexity." What is now the reply? Not, "if you will give me five hundred dollars, I will impart the desired information," but he promptly replies, that "if he had been so fortunate as to have acquired any more perfect method of performing *any* of the various operations in dental surgery, than he (Mr. A.) possessed, it would afford him the greatest pleasure to impart to him the secret of his success."

We need not carry this narration farther, the object for which it is introduced, and the result being fully apparent. Nor need we spend much time in analyzing either the motive which prompted the attitude assumed by Mr. B. at the first interview, or what had caused such "a change to come over the spirit of his dreams." It is sufficient for our purpose to observe, that although Mr. A. had procured testimonials of character and skill, yet they were given by those by no means capable of judging in regard to the proper qualifications of a *dentist*. The result was, that such testimony, (notwithstanding it was given by three highly intelligent and respectable men; the one, then a distinguished member of the state legislature, another an eminent *physician*, and the third a clergyman,) failed to remove that feeling of jealousy and distrust, which had been one of the leading precepts of that school, in which he had been educated. But, on the other hand, when he met Mr. A., as a fellow-member of an association whose object was to better the condition, by elevating the standard of his own profession, and knew well, that such membership could only have been secured by the most ample proof of his worthiness, and that given to *those eminently qualified to estimate its true value*, every ground of suspicion was removed. But this is by no means the extent of the influence imparted by this association, as exemplified by this case. It has not merely uprooted prejudice, but planted and cultivated in its stead, a brotherly regard and interest in the welfare of his fellow, which had never before existed in the profession. If this example may be regarded any criterion, and we have no doubt that it might be multiplied to almost any extent, we can but hail the establishment of this society as a new and proud era in the history of dental science. Its leading advantages, especially to its younger members, may be thus enumerated.

1st. It greatly augments their means of improvement, by becoming, at the annual conventions, personally acquainted with eminent men in the profession, and their modes of operating.

2d. Its diploma furnishes the best, nay the only *universal* passport, to the confidence of every *member of the profession* whether he is, or is not a member of the Society.

3d. When this Society shall have become fully understood by the community at large, it will be the most perfect guarantee which he could offer to the *public*, of his meriting their confidence and patronage, and will secure to them in turn, a perfect protection against imposition.

4th. As a necessary consequence, empiricism would cease to be patronised, and honest enterprise would be encouraged; placing, thus, the dental profession on that firm basis which its intrinsic value justly demands.

5th. It furnishes the means by which every honorable dental surgeon may do much more to effect the desirable reformation, than he could accomplish by any *individual* effort he could put forth.

But while we may thus speak of the benefits which *may* be derived from this association, we would by no means enumerate them as *necessary* consequences, resulting from mere membership. Nay, on the other hand, unless each individual member makes proper effort to avail himself of these benefits, they will be to him wholly lost, and the society must, in turn, regard him as an unprofitable member—one only calculated to benumb its energies, and paralyze its efforts. This leads us to consider some of the *duties* as well as the *privileges* of members.

Although these are inseparably blended, as regards the whole society, it will not necessarily hold true in respect to individual members. For example, were *every* member to neglect the duties which the constitution and a just regard for its interest, makes binding, it must follow that all benefit to be derived from it would cease, as would indeed its existence, yet *some* members might wholly disregard every rule, and still to some extent, enjoy the benefits of its influence. Such special advantage, however, must be very limited.

If, then, not only his own advance must be curtailed, but the influence of the society itself crippled, by his negligence, we would fain persuade all, that it is not only their *interest*, but their *duty*, to put forth every reasonable effort to promote the welfare and perpetuity of the American Society.

We might enumerate many ways in which these might be promoted by its members; but, on the present occasion, we shall mention but *one*, and this we regard of paramount importance. We allude to the personal attendance of each member upon the annual conventions. This, while it is one of the most important duties, is one most likely to be neglected, as it involves the greatest sacrifice of *time* and *money*. But no member, and especially the younger portion, can fail to be most abundantly repaid for any necessary sacrifice it will cost. It should be remembered, moreover, that unless the interest of these conventions can be kept up, the society cannot be efficiently sustained. If this view of the subject be correct, "*special pleading*" should be unnecessary to induce members, bound by a pledge, by interest, and by their professions, to perform so palpable a duty. We could wish that it were so; yet any one who has attended these meetings uniformly, must have observed that they are but too sparsely attended. He will have observed, also, that they are composed of nearly the same members each succeeding year. There are, indeed, many who would not be known as members, did not their *names* appear on the catalogue. In a conversation with one of these habitual delinquents, some six or eight months since, he summed up his excuses by saying that he was always so hurried that it was difficult for him to leave home—adding, moreover, that his practice had become so well established that he thought it *now* no great object for *him* to make the sacrifice, and, to use his own language, "feared it would not pay." While he thus tacitly acknowledged the benefits which even he *had* derived from this society, his sentiments we regard as simply another way of declaring that he cared not a whit, what became of the profession, its honor, or its dignity, could he but reap from it a rich harvest. It discovers a spirit which would destroy any profession, or any society, calculated to promote its interests. The same excuse, both in regard to hurry of business and fulness of practice, might, indeed, be made by a great majority of the members, and especially by those who have done most to sustain this association. For it may be safely asserted, that those who have attended these conventions *habitually*, have been almost uniformly those who, by their distance from the place of meeting, have been subjected to the greatest sacrifice, who are in *full* practice, and who feel as little the necessity of making capital of the influence of the society, as any men in the profession. It is not, then the drones of the society, nor those whose fame or fortune depend on its influence, who have contributed most to its resources and efficiency, but such as might, with far greater propriety, have urged this excuse for apathy. They have been of those who did not feel at liberty, nay, who disdained, to "reap where they had not sown"—to participate in the spoils of a victory, while they not only neglected but refused to share in the sacrifices necessary to secure it. Nor can we understand how *any* high-minded man can consent to do so. For a member to neglect, therefore, this important duty, is to rob himself of most valuable privileges—the society and the profession of a contribution which they have a right to demand—and his patients of that skill and knowledge which he might hence receive.

Of which of the benefits above enumerated, will he receive his full equivalent, by simply becoming a member, but never availing himself of the acquaintance, the experience, and the spur to action, which would be secured by meeting, annually, hundreds of the most enthusiastic and enlightened of the profession, and that under the most favorable circumstances—met together for the sole purpose of interchanging views upon all topics connected with their favorite profession? To say nothing of the value of the acquaintance he would there form, or the information to be derived from personal interviews with scientific and skilful men, and from the specimens of improvements there exhibited ; we say the inspiring influence of these meetings is worth an annual voyage across the Atlantic.

No man of any spirit ever attended one of them, without having every resolution to advance his own knowledge, and the good of the profession strengthened, his determination to eradicate quackery more firmly settled, his views enlarged, his resources for improvement augmented, and his ability for doing good enhanced. And the reward which he fails to find in *dollars and cents*, he receives through the feelings of gratitude expressed by those whom he is thereby the better enabled to serve.

To refuse, then, to make the small sacrifice necessary to attend these conventions, under ordinary circumstances, betrays, it seems to us, a short-sighted selfishness, by no means enviable, in whatever light it may be viewed. We have, on the one hand, the good of the profession, its honor and its dignity, all intimately connected with our own, and, on the other, our professional reputation, the only sure basis of wealth, to stimulate us on to active exertion, to promote the influence and perpetuity of the American Society of Dental Surgeons.

And we have every assurance, that for every talent we devote to this object, we shall, as professional dentists, receive *ten* in return.

Let but the *present* number of members, be as active as they might, and the effect would be astonishing, not only upon the profession, but upon the public at large.

And as a stimulus to activity, nothing can be better calculated than attending the annual meetings. Take from any association, for whatever purpose it may be formed, the stimulus furnished by their periodical conventions, or meetings, and you have taken away its main-spring, and left only an inert and useless mass of machinery. On the other hand, wherever we see such meetings of any association fully attended, we may safely infer that the cause which originated it, is prospering, and that its march is onward. Let each member of the American Society of Dental Surgeons but have the cheering, the electrifying stimulus, arising from the presence of *every other member*, at one of these meetings, and he would feel that the warfare against quackery must result in certain victory, and that the standard of his profession was already raised too high, to be reached, or defaced by the envenomed arrows of the empiric.

The influence of this society upon the dental profession and the public, thus sustained, might be aptly compared to the genial influence of the natural sun. The one is to the dissemination of science, with all its meliorating effects, what the other is to the dissemination of the necessary warmth, and the gentle shower, so grateful to every inhabitant of earth, and so necessary in supplying sustenance for both man and beast. While the sun, by its influence, raises from the ocean vapor, which by the natural current of air is carried throughout a vast extent of country, to be condensed into grateful showers, carrying with them into the earth, the noxious vapors, thus refreshing and rendering healthful the air, and at the same time watering the thirsty ground, so the members of the American Society, at its annual conventions, drink in from the accumulated ocean of knowledge, which, by the natural action of mind is disseminated, like the vapor of the cloud, throughout the country, there, like the shower, to descend with all its benign influences, carrying with it, into the earth, the pestilential breath of ignorance and empiricism.

The analogy stops not here.

This fresh and refreshing vapor, which was separated from the briny deep, by an almost incomprehensible influence, though it is spread far and wide, and apparently swallowed up by the thirsty earth, is far from being lost. It is collected into fertilizing streams, and returned to the ocean whence it came. Very similar is the American Society, with its annual conventions. Each member freely partakes of the scientific treasures from the grand reservoir of the whole society, returns with it to his own sphere of action, there freely imparts its blessings, in lessening the ills and increasing the comforts of those whom it is his privilege to serve, and who, in turn, so fortunate as to avail themselves of his thus accumulated skill.

Nor is this knowledge "like water spilt upon dry ground which cannot be gathered up," but as the rain, when it has served its destined office, is returned to the ocean, in a thousand useful streams, each member returns with his contribution to the next annual meeting, throwing it into the common fund, to be distributed, perhaps, to a portion of country less bountifully supplied with this healthful and fructifying element.

